# Protective Effects of the FOXO3 Longevity Gene on Aging-Related Diseases in the CHAMP Cohort

**DOI:** 10.1093/geroni/igaf122.2669

**Published:** 2025-12-31

**Authors:** Lovina Abdi, Richard Allsopp, David LeCouteur, Bradley Willcox

**Affiliations:** University of Hawaiʻi at Mānoa, Honolulu, Hawaii, United States; University of Hawaiʻi at Mānoa, Honolulu, Hawaii, United States; ANZAC Research Institute, Sydney, New South Wales, Australia; John A. Burns School of Medicine, Honolulu, Hawaii, United States

## Abstract

Recent findings from the Kuakini Honolulu Heart Program cohort in Hawaii suggest that longevity-associated gene variants of FOXO3 (e.g. SNP rs2802292-G allele carriers) may confer resilience against cardiometabolic diseases (CMD) such as hypertension, diabetes, and angina, in Japanese-American men. Therefore, we studied white males from the Concord Health and Ageing in Men Project (CHAMP) cohort in Australia, focusing on the protective FOXO3 G allele and its potential impact on CMD and longevity. We found that G allele carriers with angina exhibited a significantly reduced risk of mortality, although no similar protective effect was observed in individuals with hypertension or diabetes. These data support prior findings that the FOXO3 G allele may not universally protect against CMD onset, but it may delay disease onset and lessen the severity of advanced symptomatic CMD, such as angina, highlighting its critical role in promoting cardiovascular health. The protective effects of FOXO3 are likely mediated through multiple mechanisms. This gene has been associated with enhanced cellular resilience against oxidative stress, maintenance of telomere length to preserve genomic stability, and regulation of metabolic pathways essential for cardiovascular function. Furthermore, FOXO3 influences cytokine signaling, which may mitigate systemic inflammation—a key driver of CMD risk, progression and mortality. These pathways collectively support cardiovascular integrity and overall longevity. This study reinforces FOXO3’s importance as a genetic factor influencing CMD outcomes and longevity. Future studies are needed to uncover the precise molecular mechanisms and evaluate FOXO3 as a potential therapeutic target for CMD prevention and treatment.

